# CAR-T therapy for endocrine neoplasms: novel targets and combination of therapies

**DOI:** 10.3389/fendo.2025.1517525

**Published:** 2025-02-11

**Authors:** Fang Wang, Ruiqi Zhang, Zhaokai Zhou, Run Shi, Fu Peng, Yudi Xu, Shuai Yang, Zhan Wang, Pengpeng Zhang, Rui Tu, Chun Zhang, Xingchen Liu, Jun Cai

**Affiliations:** ^1^ Department of Otolaryngology-Head and Neck Surgery, Xinyang Central Hospital, Xinyang, Henan, China; ^2^ Department of Urology, First Affiliated Hospital of Zhengzhou University, Henan Joint International Pediatric Urodynamic Laboratory, Zhengzhou, China; ^3^ Department of Oncology, The First Affiliated Hospital of Nanjing Medical University, Nanjing, China; ^4^ Department of Pharmacology, Key Laboratory of Drug-Targeting and Drug Delivery System of the Education Ministry, Sichuan Engineering Laboratory for Plant-Sourced Drug and Sichuan Research Center for Drug Precision Industrial Technology, West China School of Pharmacy, Sichuan University, Chengdu, China; ^5^ Department of Neurology, The First Affiliated Hospital of Zhengzhou University, Zhengzhou, Henan, China; ^6^ Department of Lung Cancer, Tianjin Lung Cancer Center, National Clinical Research Center for Cancer, Key Laboratory of Cancer Prevention and Therapy, Tianjin’s Clinical Research Center for Cancer, Tianjin Medical University Cancer Institute and Hospital, Tianjin, China; ^7^ Department of Ultrasound, Xinyang Central Hospital, Xinyang, Henan, China; ^8^ Department of Gastroenterology, Affiliated Hospital of Nanjing University of Chinese Medicine, Nanjing, China; ^9^ Department of Gynaecology, Xinyang Central Hospital, Xinyang, Henan, China

**Keywords:** endocrine neoplasms, chimeric antigen receptor T cell, immunotherapy, combined immunotherapy, clinical trials

## Abstract

Endocrine malignancies constitute a heterogeneous tumour group with diverse biological characteristics. While typically indolent, they encompass aggressive types and presence of any metastatic sign indicates a high probability of recurrence and a diminished response to conventional therapies. Chimeric antigen receptor (CAR)-T cell immunotherapy has constituted a revolutionary advance in cancer treatment and exhibited significant potential for application in endocrine cancer. However, limited effectiveness was displayed in clinical application, which necessitates the exploration of novel modalities. Identification of specific and safe targets for endocrine cancer is the initial stage towards establishing a successful CAR-T treatment. Various therapies under investigation offer potential enhancements to CAR T cell efficacy through diverse mechanisms. Herein, we summarize recent advances in identifying targets of endocrine cancer for CAR therapy and provide an overview of combinatorial approaches.

## 1 Introduction

Endocrine malignancies are tumors driven from endocrine or neuroendocrine cells throughout the body resulting in disruptions in hormone production, that are heterogeneous in histology and biological features. They encompass tumors originating in traditionally endocrine organs, such as thyroid, adrenal, pancreatic, ovarian, mammary, and prostate glands, as well as neuroendocrine tumors (NETs) from non-endocrine organs, including gastrointestinal tract and bronchial tubes. Due to their low proliferative capacity, surgery to eradicate tumors is considered the standard therapy, and for some tumors like differentiated thyroid cancer (DTC), specific radiation therapy follows to decrease or eliminate tumor size and extend patients’ lives. Frustratingly, some cases are diagnosed at advanced stages due to lack of early symptoms, and curative therapy for metastatic endocrine tumors has been reported to be ineffective. Moreover, aggressive tumors, containing anaplastic thyroid cancer, small cell lung cancers (SCLC) and so on, are included among endocrine neoplasms owing to their heterogeneity. Generally, considering limited efficacy and potentially deleterious effects of conventional treatment methods, it is imperative that novel and efficacious alternatives be promptly investigated (1, 2).

In recent decades, immunotherapy has achieved significant advances in the field of cancer treatment, with an emphasis on inhibition of tumor progression through the alteration of relationship between tumor cells and immune system. Chimeric antigen receptor (CAR)-T cell is an adoptive cell-based therapy, that modifies autologous, patient-derived T cells to express a CAR and redirects their cytotoxic activity independent of major histocompatibility complex (MHC) (3, 4). Structurally, CARs are artificial receptors that are composed of an extracellular antigen-recognition domain, antibody-derived single-chain variable fragments (scFv), a transmembrane region, and intracellular co-stimulatory moiety placed upstream of CD3ζ and most frequently generated from 4–1BB or CD28 (5). Recognizing cognate tumor antigen by CARs ignites downstream signaling activities leading to activation of T-cells and targeted tumor lysis.

Despite effective clinical application in hematological malignancies, CAR-T therapy has demonstrated limited efficacy against solid tumors. The major hindrances against endocrine tumors manifest in the following aspects (3, 6): 1) paucity of target tumor antigens while limited on healthy tissues to avoid on-target off-tumor toxicity, 2) insufficiency of engineered cells homing and infiltration, 3) immunosuppressive tumor microenvironment (TME) impeding persistence and efficacy of CAR-T cells. Herein, this narrative review sought to summarize suitable novel specific targets of endocrine cancer for CAR therapy and combination therapy with alternative strategies to surmount these obstacles.

## 2 Promising antigens for CAR-T cell targeting of endocrine cancer

Identifying specific antigens to target endocrine cancers is the first step in establishing a successful CAR-T therapy. Theoretically, any antigen on tumor surface, such as proteins, glycolipids, and gangliosides, could potentially be targeted by CAR. However, expression of autoantigens in normal tissues results in non-targeted toxicity. Therefore, the ideal tumor target should be tumor-specific antigens (TSA) or tumor-associated antigens (TAA), and CAR-T could elicit a targeted immune response against tumor tissues while sparing surrounding normal tissues. The following section presents a comprehensive summary and detailed analysis of emerging potential CAR-T cell targets in endocrine-related malignancies.

### 2.1 DDL3

Delta-like Ligand 3 (DLL3) is a type I transmembrane protein and functions as an inhibitory Notch ligand whose deregulation contributes to tumorigenesis, progression and chemoresistance in several NETs (7). Therefore, this molecule showed diagnostic and therapeutic potential, whose applications such as molecular imaging, antibody conjugates and so on, were undergoing. DLL3-Notch interaction in SCLC is the first discovered and most extensively studied (8, 9). AMG 757, a bispecific T-cell engager, induced intratumoral activated T cell infiltration and significant regression of tumor overexpressing DLL3 (10). Chen et al. evaluate tumor-suppression activity of DLL3 as a CAR-T therapy target (11). Further study by Zhang demonstrated its high sensitivity and long-term killing potential with no tissue damage in brain or hormone-secretion function ablation of pituitary (12). AMG 119 is the first adoptive cellular therapy of DLL3, generated through autologous T cell transduction with a self-inactivating lentiviral vector based on human immunodeficiency virus (HIV)-1. Phase 1, first clinical study in relapsed/refractory SCLC patients reported preliminary anti-tumor effectiveness and a tolerable safety profile of AMG 119 (13). DLL3 overexpression is also detected in other tumors with neuroendocrine features including medullary thyroid carcinomas (14), cervical (15), gastroenteropancreatic (16) and prostate (17) and other endocrine cancers such as pancreatic cancer (18) and breast cancer (19), providing novel perspectives for CAR immune cell therapy.

### 2.2 SSTR

The cyclic peptide hormone somatostatin, primarily distributed throughout central nervous system, gastrointestinal tract and pancreas, plays a pivotal role in regulating a wide range of hormone-inhibitory functions. Moreover, it could exert suppressive effects on cell proliferation and inflammation (20). The realization of somatostatin function is mediated by somatostatin receptor (SSTR) expressed on cell surface, making it a potential therapeutic candidate. SSTRs, G protein-coupled receptors encoded by five conserved genes (SSTR1-SSTR5), were highly expressed in NETs (21). Mandriani and colleagues incorporated octreotide, a somatostatin analog, in extracellular moiety of CAR to target SSTR, which exerted a sound antitumor activity against pancreatic, intestinal and lung NET xenografts. Noteworthy, no obvious deleterious effects were observed in other organs expressing SSTR, providing a blueprint for therapeutic applications (22).

### 2.3 B7-H3

B7-H3 (CD276), belonging to type I transmembrane protein of B7 family, played as a co-stimulatory molecule in T cell response and mediated immunosuppressive TME *via* M2 macrophage (23, 24). B7-H3 exhibited aberrant upregulation in several primary tumors while restricted in normal tissues, and is associated with poor prognosis, prompting development of its immunotherapy targeting strategies (25). B7-H3 expression is elevated in prostate cancer cells especially stem cells after fractionated irradiation, demonstrating a potent strategy for radiotherapy resistance patients (26). Duan et al. devised a B7-H3 CAR-T that effectively targets human follicular thyroid cancer cells, inducing cytotoxic effects. This involved creation of a novel Fab CAR, comprising a tandemly linked combination of antigen-binding and TCR intracellular signaling domains, which recognizes tumor antigens independent of MHC and mimics innate endogenous TCR activation, effectively reducing premature T-cell depletion (27). A study conducted by Du and colleagues revealed promising application of B7-H3 CAR-T in ovarian cancer (OC), neuroblastoma and pancreatic cancer, without apparent toxicity to normal tissues (28). Moreover, a novel longevous B7-H3 CAR-T cell integrating 4-1BB costimulatory molecule using lentivirus transduction effectively inhibited triple-negative breast cancer (TNBC) and OC *in vivo* at low doses. The increase in CAR-T cell survival and proliferation was mediated by JAK-STAT signaling activation *via* trIL2RB and YRHQ motifs (29).

### 2.3 Gangliosides

Gangliosides are sphingolipids whose sugar chain is connected with at least one sialic acid. Nacetylneuraminic acid (NAc) and N-glycolyneuraminic acid (NGc) are two sialic acid variations that are most commonly found in mammals. The absence of an exon of CMAH, enzyme responsible for the transformation from NAc to NGc, results in lack of NGc in normal human tissues. However, N-glycoslylated ganglioside monosialic 3 (NGcGM3) produced from food has been found in numerous cancer cases, including OC and breast cancer. Utilizing 14F7-based CARs, Elisabetta Cribioli et al. demonstrated significant control of NGcGM3+ OC and caused no adverse reactions against healthy tissues (30). Disialoganglioside GD2, another potential target, is expressed on neuroectodermal origin, though with limited expression in other tissues. Due to its overexpression in SCLC and association with tumor proliferation and invasiveness (31), Reppel et al. evaluated efficacy of optimized GD2 CAR-T cells, which demonstrated its antitumor activity on orthotopic and metastatic models and superior performance against tumor re-challenges. Further GD2 upregulation by exposure to EZH2 inhibitor, enhanced lung tumor cells’ susceptibility to CAR-T cell-medicated cytotoxic effects (32).

### 2.4 Glycoprotein

Carcinoembryonic antigen-related cell adhesion molecule 5(CEACAM5, CEA), the cell surface glycoprotein, showed high levels of expression and is validated as an immunotherapeutic target for many tumors. However, no clinical immunotherapeutics targeting CEACAM5 are available. CEACAM5 was identified as an antigen enriched in cell surface of neuroendocrine prostate cancer (NEPC), which behaves biologically aggressively (33). A recent study developed a novel human monoclonal antibody, 1G9, which has exhibited high affinity and specificity to A3 and B3 of CEACAM5 (34). CAR-T based on 1G9 exhibited sound cytotoxicity against CEACAM5+ NEPC cells and mice treated with had prolonged survival. To note, research also indicated binding and potency of anti-CEACAM5 antibodies might be influenced by N-linked glycan present at A3B3 domains, indicating alterations to these glycans in tumors could potentially affect its activity. Additionally, NCAM-1 (CD56) is a glycoprotein overexpressed on surface of neuroendocrine cancers, including SCLC and neuroblastoma. Administration of sleeping beauty transposon-generated CD56-CAR T cells significantly reduced tumor burden, however, impact on survival was modest and further examination was required to ascertain potential for on-target, off-tissue effects (35).

### 2.5 Target for aberrant glycosylation

Glycolipid alterations generated by abnormal glycosylation were discovered on surface of cancer cells and their stem cells and demonstrated targeting potential (36). Stage-specific embryonic antigen-4 (SSEA-4) is a glycosphingolipid expressed on embryonic stem cells and disappearing after differentiation, which manifests in parallel with that in cancer development and progression suggesting it is a cancer stem cell marker (37). Using pancreatic cancer cell lines, targeting SSEA-4 yielded efficacious results in killing cancer cells, both *in vitro* and *in vivo (* 38). However, although adoptive transfer of SSEA-4-CAR-T cells into TNBC mice model exerted a significant tumor burden reduction, vital cell elimination with stem properties in bone marrow and lungs was observed (39). Moreover, Monzo et al. conducted a safety study that also found dose-related on-tumor off-target toxicities (40). Collectively, these researches suggested further managements of anti-SSEA-4 CAR-T toxicity were warranted.

MUC1 is a transmembrane protein of mucin family and comprises an extracellular domain of serine and threonine residues for O-glycosylated where posttranslational modifications occurred. Tumour-associated MUC1 with aberrant glycosylation is over-expressed in OC and TNBC and acts essentially in tumor metastasis and progression (41, 42). In a TNBC xenograft model treated by second-generation tMUC1-CAR-T cells, significant tumor cytolytic activity and increased Th1 type cytokine and chemokine production have recently been demonstrated, while normal breast epithelial cells were left unaffected (43). Similarly, TNBC has high expression of TnMUC1, another aberrant glycoform of MUC1 considered to be the bonafide target. Further studies revealed Tn-MUC1 was a valuable target of CAR-T cell treatment for pancreatic and ovarian cancer (44, 45). Overall, because of its high tumor antigen specificity and being masked in normal tissues, abnormal glycosylation showed a promising therapeutic avenue for CAR-T cell treatment. Clinical trials of engineered T cells targeting these glycoforms are underway (NCT04020575 and NCT04025216).

### 2.6 Miscellaneous targets

DTC makes up vast majority of thyroid cancers and is biologically indolent tumors; considered to have a long-term prognosis after surgical interventions. Unfortunately, relapse and metastases occurred and eventually led to refractory to radioactive iodine and poor survival. As patients have previously undergone thyroidectomy before other treatment regimens, concerns about CAR-T targeting healthy thyroid glands are unwarranted. Target antigen selection should not be constrained by TSAs and TAAs. Thyroid stimulating hormone receptor (TSHR), a well-known thyroid-specific antigen coexpressed in both DTC tissues and normal thyroid gland, was presented as a new candidate for CAR-T treatment without evident toxicity in a recent study (46). A clinical instance of TSHR-CD19 dual-targeted CAR-T cells being used to treat recurrent refractory thyroid cancer was documented. Dynamic monitoring of the patient revealed a potent expansion of CAR-T cells *in vivo*. The efficacy evaluation of thyroid cancer treatment achieved partial remission and remained until the patient died of pulmonary infection. Overall, these results indicate promising efficacy of TSHR+CD19 CAR-T with close monitoring of adverse effects (47). Clinical trials with adequate anticipation should investigate anti-TSHR CAR-T as a potential treatment option for individuals with local recurrence or distant metastasis of thyroid cancer.

TAAs on cells with irreplaceable functions are generally considered unsuitable for CAR development. To overcome this limitation, Feng et al. used a phage display screening method and isolated a llama-derived nanobody, VHH1, that preferentially binds to surface adhesion protein CDH17 overexpressed in NET cells (48). VHH1-CAR-T cells (CDH17-CARTs) eradicated CDH17-expressing NETs, such as gastric, pancreatic and colorectal cancer cells. To note, CDH17-CAR-Ts cause no toxicity by attacking normal intestinal epithelial cells expressing CDH17. Underlying mechanism is unique location of CDH17 at tight junction between normal intestinal epithelial cells and thereby ‘masked’ in healthy tissues, while cancer cells’ lack of polarity exposes CDH17 expression enhancing susceptibility to CAR-T-mediated killing. Therefore, CDH17 represents a class of TAA for cancer immunotherapy previously unappreciated, revealing novel insight into targeting selection.

## 3 Combination of therapies

**Figure 1:**
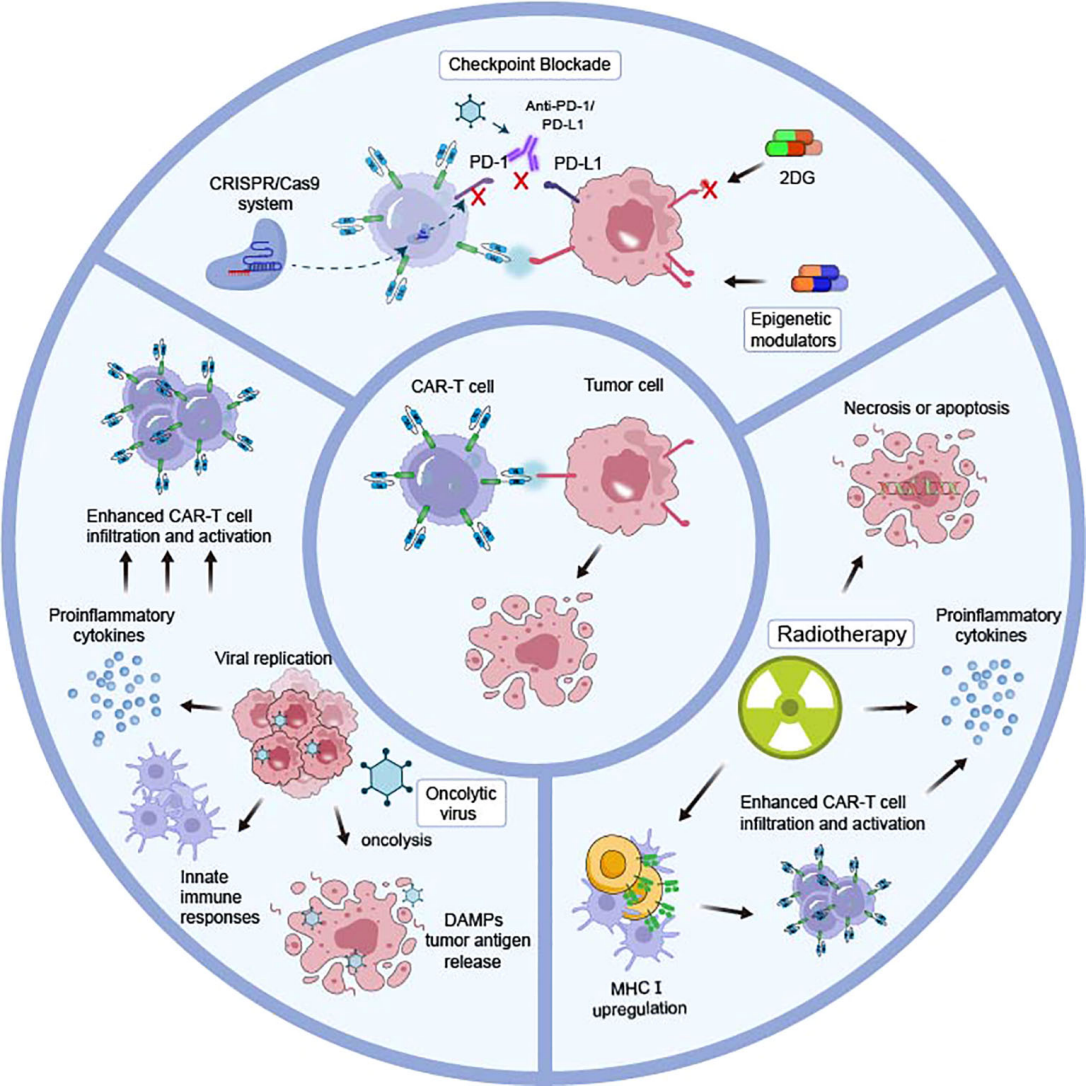
Combinatorial strategies of different mechanisms to enhance CAR-T cell efficacy in endocrine tumors. The combination of various therapeutic modalities, operating through disparate mechanisms, with CAR T cell therapy represents a promising avenue for clinical advancement. The PD-1-PD-L1 axis, which impedes CAR-T cell efficiency, could be blocked through anti-PD-1/PD-L1, oncolytic viruses, and genetic blocking by the CRISPR/Cas9 system. An augmentation of antigen expression may be achieved through the utilization of epigenetic modulators, which disrupt the N-glycan coating by 2DG. Radiotherapy triggers tumor cell necrosis or apoptosis, provides a favorable tumor microenvironment by upregulation of MHC class I, and enables CAR-T cells to infiltrate and increase IFNγ production, thus enhancing infiltration cytotoxicity of engineered T cells. Oncolytic viruses replicate selectively in tumor cells and could be genetically modified to produce various therapeutic agents that enhance CAR-T cell infiltration and activation.

Endocrine tumors are predominantly indolent, and for foreseeable future, monotherapy of CAR-T cells is unlikely to supplant conventional therapeutic strategies, but would be integrated into combination therapies. Therefore, it is insufficient to focus solely on optimization of CAR structure. In addition, immunotherapy is mostly used as salvage therapy, so it is anticipated to be confronted with more aggressive and rare subtypes, and the use of combination treatments targeting multiple mechanisms simultaneously has proven potential as a strategy for cancer treatment (**Figure 1**).

Radiotherapy is integral to many endocrine tumors’ standard treatment and could be implemented as a CAR conditioning regimen. Enhanced cytotoxicity of engineered T cells by radiotherapy might be achieved through the following (49–52): 1) The DNA-damaging properties of ionizing radiation kill tumor cells and trigger pressure signals; 2) Sensitivity to apoptosis of radiation-treated cancer cells was enhanced; 3) A favorable tumor microenvironment is provided by upregulation of MHC class I, which enables CAR-T cells infiltration and increases IFNγ production. In a recent study, treatment of fractionated irradiation significantly upregulates B7-H3 expression in prostate cancer cells, and is more effective in controlling xenograft growth in mice after B7-H3 CAR-T-cell administration than monotherapies (26). The approach presented provides a sound basis for multimodality CAR therapy with radiotherapy conditioning for eliminating clonally heterogeneous tumors. However, dose and frequency of radiotherapy and sequence of this unique combinatorial model warranted further clinical examination and optimization for improving efficacy while minimizing toxicity.

Additionally, considerable research has also been done to investigate pharmaceutical methods for stimulating expression of targeted antigens (53). In neuroblastoma GD2, while exhibiting uniform expression, could be further elevated after treatment with histone deacetylase inhibitor vorinostat *in vitro (* 54). Reppel L et al. delineated that GD2 could be upregulated in GD2 SCLC cell lines after exposure to Enhancer of Zeste Homolog (EZH2) inhibitor tazemetostat, rendering superior sensitivity to GD2-CAR-T cells, which demonstrated that CAR-T cell therapy with selected epigenetic modulators deserve further preclinical investigation to minimize antigen escape (32).

Aberrantly glycosylated alterations provide specific markers employed in immunotherapeutic strategies. In opposite scenarios, target antigens after post-translational modifications might raise CAR-T cell activation threshold. N-glycans were proven to provide multifaceted protection to pancreatic adenocarcinoma cells by lytic immune synapse formation and interfering with transcriptional activation, cytotoxicity, and cytokine production through inhibiting N-glycan synthesis by knocking out mannoside acetyl-glucosaminyltransferase 5 (MGAT5) (55). Prophylactic administration of glucose analog 2-deoxy-d-glucose (2DG), which preferentially accumulates in tumors with high metabolic demand, offsets glycan coat and restores susceptibility to CAR-T cell. Moreover, 2DG treatment impairs proper functionality of PD-1-PD-L1 axis and leads to reduced immunosuppression. The combined 2DG and CAR-T cell therapy demonstrated efficacy against a range of carcinomas, including those arising from lung, ovary, and bladder, and with diverse clinically relevant CAR targets, such as CD44v6 and CEA. Overall, deglycosylating strategies have immediate translational opportunities and proved to be a viable approach to rational design of adjunct therapy for CAR-T cells.

Immune checkpoint inhibitors (ICIs) are agents which function through impeding immune evasion mechanisms exhibited by various cancers, and programmed cell death 1 (PD-1) and its ligand (PD-L1) blockers are representative ICIs. A significant increase in expression of PD-1 on CAR-T cells was observed in a Her2^+^ mouse model which contribute to impairing efficiency (56). Therefore, a combined immunotherapeutic approach of CAR-T cell therapy and PD-1 inhibition was hypothesized to overcome PD-1/PD-L1 pathway-mediated tumor immunosuppression and restore engineered cells’ ability. However, advancing in clinical experience is in its early stages, and initial findings are promising in some respects, yet in some cases, results are less encouraging (57, 58). In a clinical trial (NCT01822652) of GD2-CAR T cells for neuroblastoma, addition of PD-1 blockade showed no measurable effect on enhancing outcomes (59). The mechanisms through which checkpoint blockade exerts its beneficial effects on CAR-T persistence remain to be elucidated. It has been demonstrated that CAR-T cell modification through knockout of the PD-1-encoding gene, PDCD1, represents an alternative strategy for blocking the pathway without systemic toxicity (60).

Another study demonstrated oncolytic virus (OV) is another attractive approach for blocking PD-L1 signaling (61). OVs are capable of selectively infecting and lysing tumour cells while sparing healthy cells. In addition, they could genetically be modified to produce a range of therapeutic agents that facilitate tumour antigen presentation, stimulate innate immune responses and thus turn immunosuppressive milieu into a stimulatory for potentiating adoptive cell therapy (62, 63). *In vivo* and *in vitro* experiments of prostate cancer revealed that coadministration of oncolytic adenovirus expressing PD-L1 mini-antibody and CAR-T cells effectively promoted effector cell proliferation, resulting in enhanced anti-tumor efficacy and extended tumor-bearing mice survival. A previous study also presented that intratumoral release of chemokine, RANTES and IL15, expressed by OVs could facilitate migration and survival of adoptively transferred GD2-CAR-T cells, which results in superior anti-tumor effects in a neuroblastoma xenograft model (64). Preclinical studies support potential for further exploration of this combinatorial approach and clinical trials for preliminary safety and efficacy evaluation are recruiting (NCT03740256 and NCT05057715).

## 4 Prospect and conclusion

There is an unmet medical need to development of effective treatments for heterogeneous endocrine tumors. CAR-T therapy is gradually progressing, and researchers are engaged in developing novel strategies to overcome barriers of lack of efficacy and safety. Paucity of specific and safe neoantigens is the major obstacle hindering pursuit of CAR-T development. The review presents promising targets and current clinical trials (**Table 1**), correspondingly CAR structure could be further optimized. Targeting two or more antigens instead of one reduces possibility of antigen loss or mutation and guarantees consistent immune activation of CAR cells. In fact, there are two principal varieties of bispecific CAR-T. TAG-72 and CD47 CAR-T designed by Shu et al. which express two scFV on two CAR proteins, could inhibit the OC development, and resist drug resistance caused by OC antigens down-regulation or expression of different antigens (65). The other is to express two scFV on the same CAR protein. Liang et al. observed an increased proliferation rate of tandem CAR-T targeting FOLR1 and MSLN, demonstrating a robust anti-tumour effect and significantly prolonged survival (66). Additionally, CAR-related endocrine tumor therapy has been extended from T cells to NK cells and macrophages. The recognition of target cells by NK cells is MHC-independence indicating the low risk of NK-cell allo-transplantation. Moreover, NK cells are not prone to cause cytokine storm due to different cytokine profile from T cells and exhibit less non-targeted toxicity with limited survival time (67). Preclinical research has confirmed the potent anti-tumour efficacy of CAR-M, which targets tumour cells based on the phagocytic activity of macrophages while enhancing antigen presentation and influencing the M1 polarization of tumour-associated macrophages. Objectively, CAR-NK and CAR-M are likely to provide breakthroughs and have intrinsic benefits over CAR-T in solid malignancy treatment, while their wide application is still limited and warrants further investigation (68).

**Table 1:** Current CAR-T clinical trials of targets discussed for endocrine neoplasms.

NCT Number	Study Status	Conditions	Target	Interventions	Phases
NCT04670068	RECRUITING	Epithelial Ovarian Cancer	B7-H3	DRUG: CAR.B7-H3|DRUG: Fludarabine|DRUG: Cyclophosphamide	PHASE1
NCT04897321	RECRUITING	Neuroblastoma	B7-H3	DRUG: Fludarabine|DRUG: Cyclophosphamide|DRUG: MESNA|DRUG: B7-H3 CAR T cells	PHASE1
NCT05143151	UNKNOWN	Advanced Pancreatic Carcinoma	B7-H3	BIOLOGICAL: CD276 CAR-T cells	PHASE1|PHASE2
NCT05211557	RECRUITING	Ovarian Cancer	B7-H3	BIOLOGICAL: fhB7H3.CAR-Ts	PHASE1|PHASE2
NCT06158139	RECRUITING	Pancreas Cancer	B7-H3	BIOLOGICAL: iC9-CAR.B7-H3 T cell infusion	PHASE1
NCT06305299	RECRUITING	Ovarian Cancer	B7-H3	BIOLOGICAL: iC9-CAR.B7-H3 T cells|DRUG: Cyclophosphamide|DRUG: Fludarabine	PHASE1
NCT06646627	NOT_YET_RECRUITING	Ovarian Cancer	B7-H3	DRUG: B7-H3CART	PHASE1
NCT06055439	RECRUITING	Neuroendocrine Tumors|Colorectal Cancer|Gastric Cancer	CDH17	BIOLOGICAL: CHM-2101 CAR-T cells	PHASE1|PHASE2
NCT06501183	RECRUITING	CDH17-positive Advanced Malignant Solid Tumors	CDH17	BIOLOGICAL: Anti-CDH17 CAR-T cells	PHASE1
NCT02850536	COMPLETED	Liver Metastases	CEA	BIOLOGICAL: anti-CEA CAR-T cells	PHASE1
NCT03818165	TERMINATED	Metastatic Pancreatic Carcinoma	CEA	BIOLOGICAL: CAR2 Anti-CEA CAR-T cells	PHASE1
NCT04348643	UNKNOWN	Solid Tumor|Lung Cancer|Colorectal Cancer|Liver Cancer|Pancreatic Cancer|Gastric Cancer|Breast Cancer	CEA	BIOLOGICAL: CEA CAR-T cells	PHASE1|PHASE2
NCT06006390	RECRUITING	Gastric Cancer|Colon Cancer|Rectal Cancer|Esophageal Cancer|Pancreas Cancer|Lung Cancer|Breast Cancer	CEA	BIOLOGICAL: CEA-targeted CAR-T cells|BIOLOGICAL: CEA-targeted CAR-T cells	PHASE1|PHASE2
NCT06010862	RECRUITING	Gastric Cancer|Colon Cancer|Pancreas Cancer|Cholangiocarcinoma|Lung Cancer|Breast Cancer	CEA	BIOLOGICAL: CEA CAR-T cells|BIOLOGICAL: CEA CAR-T cells	PHASE1
NCT06043466	RECRUITING	Colorectal Cancer|Esophagus Cancer|Gastric Cancer|Pancreas Cancer|Breast Cancer|Bile Duct Cancer	CEA	BIOLOGICAL: CEA-targeted CAR-T cells	PHASE1
NCT06126406	RECRUITING	Gastric Cancer|Colon Cancer|Rectal Cancer|Breast Cancer|Lung Cancer |Pancreas Cancer	CEA	BIOLOGICAL: CEA-targeted CAR-T cells|BIOLOGICAL: CEA-targeted CAR-T cells	PHASE1
NCT03267173	UNKNOWN	Pancreatic Cancer	CEA, MUC1	DRUG: Chimeric antigen receptor T cell	EARLY_PHASE1
NCT01822652	ACTIVE_NOT_RECRUITING	Neuroblastoma	GD2	GENETIC: iC9-GD2 T Cells - frozen|GENETIC: iC9-GD2 T Cells - fresh|DRUG: Cytoxan|DRUG: Fludara|DRUG: Keytruda|GENETIC: iC9-GD2 T cells	PHASE1
NCT03356795	UNKNOWN	Cervical Cancer	GD2	BIOLOGICAL: Cervical cancer-specific CAR-T cells	PHASE1|PHASE2
NCT03373097	RECRUITING	Neuroblastoma	GD2	BIOLOGICAL: GD2-CART01	PHASE1|PHASE2
NCT03635632	ACTIVE_NOT_RECRUITING	Neuroblastoma	GD2	GENETIC: C7R-GD2.CART cells	PHASE1
NCT03721068	RECRUITING	Neuroblastoma	GD2	BIOLOGICAL: iC9.GD2.CAR.IL-15 T-cells|DRUG: Cyclophosphamide|DRUG: Fludarabine	PHASE1
NCT04430595	UNKNOWN	Breast Cancer	GD2	BIOLOGICAL: 4SCAR T cells	PHASE1|PHASE2
NCT04539366	RECRUITING	Neuroblastoma	GD2	PROCEDURE: Biopsy|PROCEDURE: Biospecimen Collection|DRUG: Cyclophosphamide|PROCEDURE: Echocardiography|DRUG: Fludarabine Phosphate|BIOLOGICAL: GD2-CAR-expressing Autologous T-lymphocytes|PROCEDURE: Imaging Procedure|PROCEDURE: Magnetic Resonance Imaging of the Heart|PROCEDURE: Multigated Acquisition Scan	PHASE1
NCT05437315	RECRUITING	Solid Tumor	GD2	BIOLOGICAL: bi-4SCAR GD2/PSMA T cells	PHASE1|PHASE2
NCT05437328	RECRUITING	Malignant Disease	GD2	BIOLOGICAL: bi-4SCAR GD2/CD56 T cells	PHASE1|PHASE2
NCT05438368	RECRUITING	Cancer Disease	GD2	BIOLOGICAL: bi-4SCAR GD2/CD70 T cells	PHASE1|PHASE2
NCT05990751	RECRUITING	Neuroblastoma	GD2	BIOLOGICAL: GD2 CAR T cells	PHASE1
NCT04420754	RECRUITING	Anaplastic Thyroid Cancer|Relapsed/Refractory Poorly Differentiated Thyroid Cancer	ICAM-1 and SSTR2	BIOLOGICAL: AIC100 CAR T Cells	PHASE1
NCT02587689	UNKNOWN	Hepatocellular Carcinoma|Non-small Cell Lung Cancer|Pancreatic Carcinoma|Triple-Negative Invasive Breast Carcinoma	MUC1	BIOLOGICAL: anti-MUC1 CAR T Cells	PHASE1|PHASE2
NCT06469281	RECRUITING	Epithelial Ovarian Cancer|Primary Peritoneal Carcinoma|Fallopian Tube Cancer	MUC16	OTHER: 27T51|DRUG: Cemiplimab|DRUG: Bevacizumab	PHASE1

The combination of CAR-T cell therapy with other therapeutic modalities offers considerable promise and encompasses a multitude of potential avenues for exploration (69). It is challenging to determine which strategy should be prioritized over another, given the multitude of processes and advantages associated with different combinations. To fully realize potential of combinatorial CAR-T cell therapy in cancer treatment, further research must be conducted in both preclinical and clinical settings. Furthermore, additional issues require resolution, including optimization of dosage regimens, minimization of additive toxicities, and the identification of predictive biomarkers (4, 70). The future direction of research will be informed by knowledge gathered from ongoing clinical studies, which will be crucial in assessing long-term results, safety, and effectiveness of CAR-T cell combination therapies.

Taken together, continued momentum of this field would depend on engineering CAR-T cells to attain specificity, safety, and efficacy, as well as selection of optimal combination therapies to enhance clinical outcomes in patients with endocrine-related malignancy.
